# Construction and validation of a cuproptosis-related lncRNA signature as a novel and robust prognostic model for colon adenocarcinoma

**DOI:** 10.3389/fonc.2022.961213

**Published:** 2022-07-28

**Authors:** Miaorong Xu, Jiayi Mu, Jiaojiao Wang, Qin Zhou, Jianwei Wang

**Affiliations:** ^1^ Department of Colorectal Surgery and Oncology, Key Laboratory of Cancer Prevention and Intervention, Ministry of Education, The Second Affiliated Hospital, Zhejiang University School of Medicine, Hangzhou, China; ^2^ Department of Colorectal Surgery, 4th Affiliated Hospital, Zhejiang University School of Medicine, Yiwu, China

**Keywords:** cuproptosis, colon adenocarcinoma, lncRNA, immune microenvironment, prognosis prediction

## Abstract

**Background:**

Cuproptosis, a newly identified form of programmed cell death, is thought to play a role in tumorigenesis. Long non-coding RNAs (lncRNAs) are reported to be associated with tumor progression and prognosis in colon adenocarcinoma (COAD). However, the role and prognostic value of cuproptosis-related lncRNAs in COAD remains unknown. This study is devoted to constructing and validating a cuproptosis-related lncRNA signature that can predict COAD patient outcomes using bioinformatics methods.

**Methods:**

The COAD mRNA and lncRNA expression profiles and corresponding clinical data were downloaded from The Cancer Genome Atlas (TCGA) database and 2,567 cuproptosis-related lncRNAs were obtained. A 10 cuproptosis-related-lncRNA prognostic signature was then constructed using the least absolute shrinkage and selection operator (LASSO) algorithm and Cox regression model and patients were divided into high- and low-risk groups. Kaplan-Meier analysis, receiver operating characteristic (ROC) curve, and a nomogram were employed to evaluate the predictive power of the signature. The immune characteristics and drug sensitivity were also investigated based on the signature. Quantitative reverse transcription polymerase chain reaction (qRT-PCR) was performed to verify the risk model. *In vitro* experiments were conducted to validate the expression of the ten lncRNAs during cuproptosis.

**Results:**

The high-risk group was associated with shorter overall survival (OS) time in COAD patients (p<0.001). Multivariate Cox regression indicated that a high-risk score was an independent risk factor for poor prognosis (p<0.001). ROC curve analysis was performed to confirm the validity of the signature (area under the curve (AUC) at 3 years: 0.879). Gene Ontology (GO) enrichment analysis revealed that the signature was highly correlated with the immune response in biological processes. The immune function, the score of the immune cells, and the expression of immune checkpoints were significantly different between the two risk groups. Three drugs, LAQ824, FH535, YM155, were found to be more sensitive in the high-risk group. Finally, the expression levels of the ten lncRNAs comprising the signature were tested by qRT-PCR.

**Conclusion:**

A ten-cuproptosis-related lncRNA signature was constructed that provided promising insights into personalized prognosis and drug selection among COAD patients.

## Introduction

Colon adenocarcinoma (COAD) is one of the most prevalent malignant tumors in humans and is associated with both high morbidity and mortality (1). In 2022, an estimated 151,000 individuals were newly diagnosed with COAD in the United States, and 52,580 succumbed to the disease (1). In 2020, COAD was associated with approximately 1.9 million cases and 0.9 million deaths worldwide (2). While the overall incidence rate has declined as a result of increased colonoscopy screening, incidence rates among people<50 years of age have increased since the end of the last century (3). Despite many advances in diagnostic and therapeutic strategies, COAD patient survival remains low (4). A previous study predicted that COAD deaths will increase substantially before 2035 (5). Thus, it is critical to identify biomarkers for COAD to help guide individualized prognostics and therapeutics.

Copper is an essential micronutrient with both beneficial and detrimental functions. It is reported that copper could promote cell proliferation through the activation of RAS-RAF-MEK-ERK signaling cascade (6). Copper is also able to activate many angiogenic factors, thus promote tumor progression and metastasis (7). Cuproptosis is a mechanism of cell death recently identified by Tsvetkov et al. that is mediated by the direct binding of copper to lipoylated components of the tricarboxylic acid (TCA) cycle. This binding leads to lipoylated protein aggregation and the consumption of iron-sulfur cluster proteins, resulting in proteotoxic stress and ultimately cell death (8). The mechanisms of this copper ionophore-induced cell death differ from other types of programmed cell death, including ferroptosis, necroptosis, oxidative stress, apoptosis, and autophagy (8). Interestingly, FDX1 and lipoylated proteins, the key regulators of cuproptosis, are highly correlated across a diversity of human tumors. Cell lines with high lipoylated protein levels are sensitive to copper-induced cell death, suggesting that copper ionophores may serve as a potential treatment for tumors with these metabolic characteristics (8).

Long-noncoding RNAs (lncRNAs) are RNA transcripts that are >200 nucleotides in length and have little or no protein-coding capacity. They play an indispensable role in regulating the expression of various cancer-associated genes (9). LncRNAs are thought to regulate genes by affecting translation, histone modification, and post-transcriptional processes (10). They are also shown to play an important regulatory role in the cell cycle, cell differentiation, and tumor development (11). Recently, several molecular signatures, especially those involving lncRNA, were developed as novel prognostic indicators of survival in patients with cancer (12, 13). However, the role of lncRNAs in regulating cuproptosis during COAD remains unknown. The value of cuproptosis-related lncRNAs as prognostic biomarkers for COAD patients has never been systematically evaluated.

The current study screened cuproptosis-related lncRNAs in COAD patients to construct and validate a novel prognostic signature. The signature performed well in immune characteristic classification and drug selection.

## Materials and methods

### Data collection

Original transcriptome data from 473 COAD tissues and 41 normal colon tissues and the corresponding clinical data were downloaded from TCGA database (https://portal.gdc.cancer.gov/). Age, gender, pT stage, pN stage, pM stage, AJCC stage, survival status, and survival time were included in the clinical data while grade was excluded because all patients were classified as unknown. A total of 446 patients with both clinical and sample information were finally enrolled and randomly divided into a training set (n=223) and a validation set (n=223) using the R “caret” package.

### Identification of cuproptosis-related lncRNAs

To identify cuproptosis-related lncRNAs, 19 cuproptosis-related genes were retrieved through a literature review (**Supplementary Table S1**) (7, 8, 14). Pearson’s correlational analysis was implemented to investigate the correlation between cuproptosis-related genes and lncRNAs, and cuproptosis-related lncRNAs were identified using the Pearson correlation coefficient and p values. A Pearson correlation coefficient >0.4 and p<0.001 were considered statistically significant.

### Principal component analysis

Principal Component Analysis (PCA) is a widely used tool for dimensionality reduction and feature extraction in the computer vision field (15). The R “scatterplot3d” package was used to assess potential differences between the high- and low-risk groups.

### Functional enrichment analysis

GO analysis covered three domains: biological processes (BP), cellular components (CC) and molecular functions (MF). Using the GO database, the biological functions of the target genes were obtained for CC, MF, and BP (16). The Kyoto Encyclopedia of Genes and Genomes (KEGG) (https://www.kegg.jp/), a database that integrates genomic, chemical, and system functional information, is commonly used for biological pathway information analysis (17). Both GO and KEGG analyses were performed using the R clusterProfiler package. The R “gsva” package was utilized to calculate the infiltrating score of 16 immune cells and the activity of 13 immune-related pathways based on a single-sample gene set enrichment analysis (ssGSEA) (18).

### Establishment of the risk signature

The prognostic values of each lncRNA were first evaluated using univariate Cox regression analysis and 318 prognostic cuproptosis-related lncRNAs were obtained. Least Absolute Shrinkage and Selection Operator (LASSO) regression was then employed to select predictors and avoid overfitting. Multivariate Cox regression analysis was then performed to identify the final candidates involved in the risk signature. A risk signature was constructed based on cuproptosis-related lncRNAs to predict COAD patient outcomes. The risk score was calculated as follows:


$$\text{Risk Score}=\sum_{i=1}^{N}\text{(Expi}*\text{Coei)}$$

N represents the number of prognostic cuproptosis-related lncRNAs in the risk signature, Expi represents the expression value of each lncRNA, and Coei represents the regression coefficient of each lncRNA in the multivariate Cox regression analysis. According to the median risk score, the patients were divided into high- and low-risk groups.

### Construction of the nomogram

R “survival” and “RMS” packages were used to construct a nomogram that combined the risk score with the clinicopathological characteristics to predict and analyze the 1, 3, and 5-year survival of COAD patients. A calibration curve was employed to test whether the predicted survival rate was consistent with the actual survival rate.

### Drug sensitivity analysis

The drug sensitivity of patients in different risk groups was evaluated using the R “pRRophetic” package, which could predict the 50% inhibiting concentration (IC50) of common chemotherapeutic drugs for colon cancer. The difference between groups was assessed using the Wilcoxon signed-rank test.

### Cell culture

DLD1 and HT29 were cultured in RPMI-1640, and HCT116 and SW480 were cultured in Dulbecco’s modified Eagle medium. Both media were supplemented with 100 U*mL^-1^ penicillin and streptomycin as well as 10% fetal bovine serum in a humidified atmosphere of 5% CO2 at 37°C.

### Reagents and drug treatment *in vitro*

Copper ionophore elesclomol was purchased from Selleck. Copper chloride was obtained from Sangon. When cells were adherent and had morphologically spread, cells were treated with 2μM copper chloride or 20nM elesclomol for 24h. After treatment, cells were harvested, and RNA was collected *via* the following extraction method.

### RNA extraction and quantitative real-time polymerase chain reaction

Eleven pairs of colon cancer and adjacent tissue samples were collected from the Second Affiliated Hospital of Zhejiang University School of Medicine. Total cellular and tissue RNA was extracted using a total RNA extraction kit (73404, Qiagen) according to standard protocol. The RNA was used to synthesize complementary DNA (cDNA) with a cDNA Synthesis SuperMix (11141ES60, Yeasen). The cDNA was used as a template and lncRNA expression was quantified with the Roche LightCycler 480 using SYBR Green Master Mix (11198ES25, Yeasen). β-actin was used as an endogenous control. Primers were synthesized by Sangon Biotech (Sangon, China). The sequences are listed in **Table 1**.

**Table 1:** The primers for 10 cuproptosis-related lncRNAs.

AC008752.2	Forward: 5’- GTGGGAGGGCATCGCTTATT -3’
	Reverse: 5’- GAAGCCCCTCCGTTTTGAAG -3’
AP001619.1	Forward: 5’- TGACTTTGAGCCCGCAGAAT -3’
	Reverse: 5’- GCTGTTGGCGTAGAGAAGGT -3’
AC020917.2	Forward: 5’- CCCCCACTTCAGGAGAATGC -3’
	Reverse: 5’- TTGGGAAAACCCTGTGGTCG -3’
AC002066.1	Forward: 5’- CTGCTACTTGGCTGATGAATGG -3’
	Reverse: 5’- CAAGCACCTAAAGCACCCCT -3’
LINC01252	Forward: 5’- TTTGATGTTCCACCCCCACC -3’
	Reverse: 5’- GACTGTTGCATCTCCTGGCA -3’
AC010789.2	Forward: 5’- AGCACAAAGTGGCAAACGTC -3’
	Reverse: 5’- TTGGGGATTGGTCAGCTTCT -3’
LINC02542	Forward: 5’- AGGGAGTCTTAAACGGAGGGA -3’
	Forward: 5’- AGGGCAGCACAGAACTGATT -3’
AC012313.5	Forward: 5’- TGTCCAGTAAACAGCCACTGA -3’
	Forward: 5’- CCTACATTGGGGTTTGAGGCT -3’
AL356804.1	Forward: 5’- GAGTCCCAACAAGGGAGAAGG -3’
	Forward: 5’- TGTGCTTTGTAGGGAAGTATCTGT -3’
ZFHX2-AS1	Forward: 5’- GGGGGTCGAATGAAAGACAGA -3’
	Forward: 5’- TCTCTCAAACCTGCACACTGA -3’
Homo β-actin	Forward: 5’- CCTTCCTGGGCATGGAGTC -3’
	Reverse: 5’- TGATCTTCATTGTGCTGGGTG -3’

### Statistical analysis

Pearson’s correlation, Cox regression, and Kaplan-Meier curve analyses were performed using R software (version 4.0.3; https://www.r-project.org/). Analysis of variance (ANOVA) was performed using GraphPad Prism software (version 9.0.0). P values<0.05 denoted statistically significant differences.

## Results

### Identification of cuproptosis-related lncRNAs with a prognostic value in COAD patients

**Figure 1:**
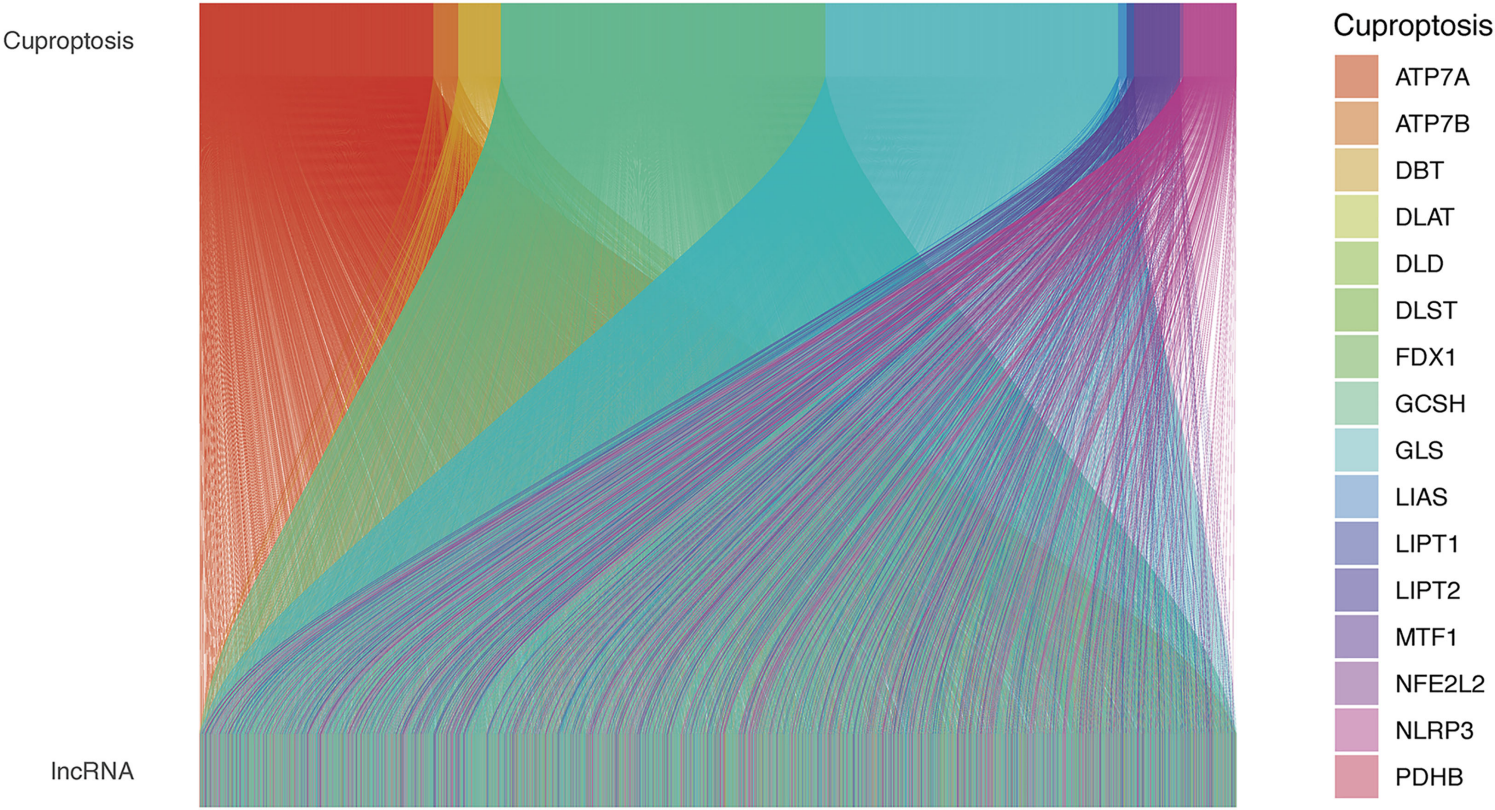
The relationships between cuproptosis-related genes and cuproptosis-related lncRNAs in the Sankey diagram. lncRNA, long noncoding RNA.

**Figure 2:**
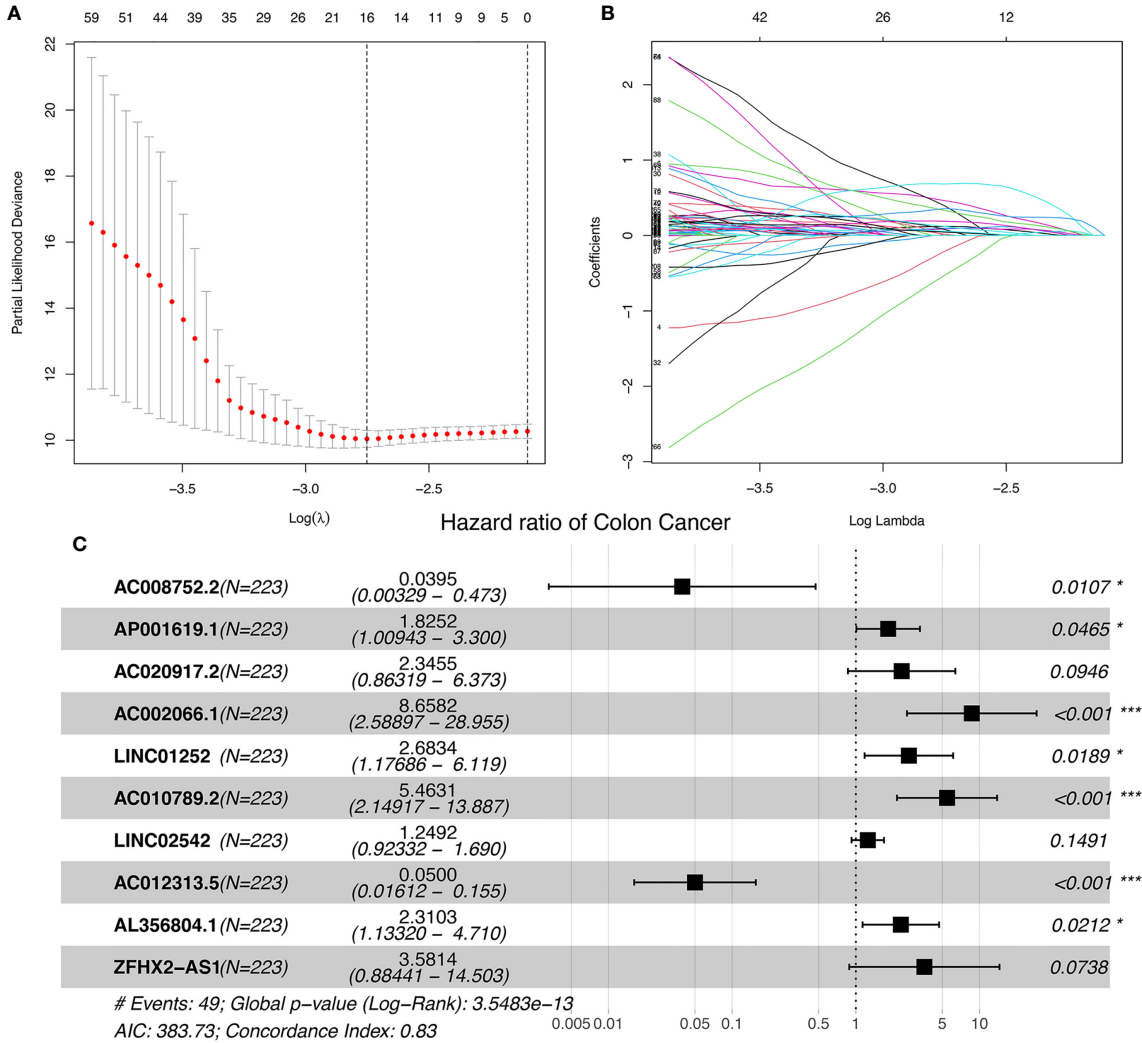
Ten cuproptosis-related lncRNAs with prognostic significance were selected. **(A)** The confidence intervals for each lambda were shown. Dotted vertical lines were drawn at the optimal values by using the minimum criteria. **(B)** Partial likelihood deviance for different numbers of variables. The horizontal axis represents the log value of the independent variable lambda, and the vertical axis represents the coefficient of the independent variable. **(C)** Multivariate Cox regression analysis showed 10 cuproptosis-related lncRNAs. nsP >= 0.05, *P < 0.05, **P < 0.01, ***P < 0.001.

The study workflow is shown in **Figure S1**. Nineteen cuproptosis-related genes and 16,876 lncRNAs were extracted from TCGA database COAD cohort. Co-expression analysis was performed to identify lncRNAs associated with cuproptosis-related genes. A total of 2,567 cuproptosis-related lncRNAs were identified with the criteria of |Pearson R| >0.4 and a p-value<0.001 (**Figure 1**) (**Tables S2**, **S3**). A total of 446 COAD patients were randomly assigned to either the training set (N = 223) or the validation set (N =223). The clinical characteristics of the samples in the two groups are shown in **Table 2**. To explore the relationship between cuproptosis-related lncRNAs and survival, univariate Cox regression analysis was performed and 318 cuproptosis-related lncRNAs with prognostic significance were obtained in the training set (**Table S4**). LASSO regression analysis was then used to select 16 cuproptosis-related lncRNAs (**Figures 2A, B**). Multivariate Cox regression analysis was used to identify prognostic cuproptosis-related lncRNAs. Eight of the ten cuproptosis-related lncRNAs, AP001619.1, AC020917.2, AC002066.1, LINC01252, AC010789.2, LINC02542, AL356804.1, and ZFHX2-AS1 with a hazard ratio (HR) >1 were found to be poor prognostic predictors while the other two, AC008752.2 and AC012313.5, may be protective indicators (**Figure 2C**). The relationships between the 10 cuproptosis-related lncRNAs and cuproptosis genes are shown in **Table 3**. These findings were supported using Kaplan-Meier survival analysis (**Figures S2A–J**).

**Table 2:** The clinical characteristics of colon cancer patients in the training and validation group.

Characteristics	Training group No.	%	Validation group No.	%	P-value
**Age**	–	–	–	–	–
<=65	81	36.32	102	45.74	>0.05
>65	142	63.68	121	54.26	–
**Gender**	–	–	–	–	–
Female	102	45.74	110	49.33	>0.05
Male	121	54.26	113	50.67	–
**AJCC Stage**	–	–	–	–	–
I	35	15.7	40	17.94	>0.05
II	89	39.91	86	38.57	–
III	64	28.7	60	26.91	–
IV	30	13.45	31	13.9	–
unknown	5	2.24	6	2.69	–
**T stage**	–	–	–	–	–
T1	6	2.69	5	2.24	>0.05
T2	35	15.7	41	18.39	–
T3	156	69.96	147	65.92	–
T4	26	11.66	30	13.45	–
**N stage**	–	–	–	–	–
N0	131	58.74	134	60.09	>0.05
N1	52	23.32	50	22.42	–
N2	40	17.94	39	17.49	–
**M stage**	–	–	–	–	–
M0	164	73.54	165	73.99	>0.05
M1	30	13.45	31	13.9	–
unknown	29	13	27	12.11	–

**Table 3:** The correlation between cuproptosis related genes and 10 lncRNAs.

CRG	LncRNAs	Cor.	P value	Regulation
GCSH	AC008752.2	0.46230763	2.02E-26	positive
GCSH	AP001619.1	0.50301926	1.06E-31	positive
GCSH	AC020917.2	0.40358817	5.90E-20	positive
GCSH	AC002066.1	0.43139939	7.33E-23	positive
GCSH	LINC01252	0.40628781	3.17E-20	positive
GCSH	AC010789.2	0.43724621	1.66E-23	positive
GCSH	LINC02542	0.48920025	7.88E-30	positive
ATP7A	AC012313.5	0.54553241	4.97E-38	positive
MTF1	AC012313.5	0.51434588	2.66E-33	positive
GLS	AC012313.5	0.44969191	6.34E-25	positive
DBT	AC012313.5	0.43715548	1.70E-23	positive
GCSH	AC012313.5	0.59341848	2.47E-46	positive
ATP7A	AL356804.1	0.45029159	5.40E-25	positive
GLS	AL356804.1	0.51339084	3.64E-33	positive
GCSH	AL356804.1	0.61840174	3.02E-51	positive
ATP7A	ZFHX2-AS1	0.41783481	2.07E-21	positive
GLS	ZFHX2-AS1	0.44742683	1.16E-24	positive
GCSH	ZFHX2-AS1	0.67499678	3.46E-64	positive

CRG, cuproptosis-related genes; Cor, the Pearson correlation coefficient.

### Construction and validation of the prognostic model

**Figure 3:**
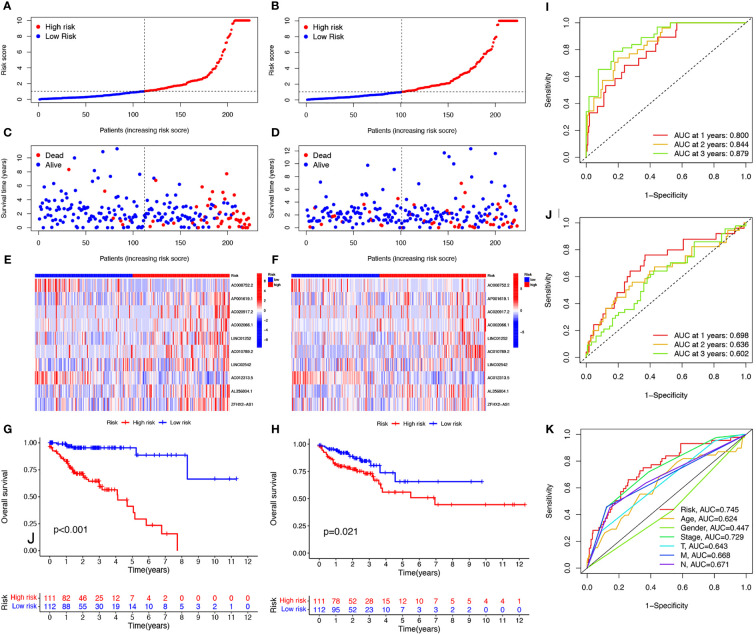
Evaluation of prognosis prediction ability of cuproptosis-related lncRNAs prognostic signature. Risk scores in the high and low-risk groups for the prognostic signature in COAD in the training set **(A)** and validation set **(B)**. Survival status of patients with COAD in high- and low-risk groups in the training set **(C)** and validation set **(D)**. Expression of the 10 cuproptosis-related lncRNAs in COAD in the training set **(E)** and validation set **(F)**. Survival analysis of the high-risk group and the low-risk group in the training set **(G)** and validation set **(H)** based on the TCGA dataset. ROC curve of the prognostic signature in the training **(I)**, validation **(J)**, and entire **(K)** sets. ROC curve, receiver operating characteristics curve; AUC, area under the curve; P<0.05, statistically significant.

To further explore the prognostic ability of the 10 cuproptosis-related lncRNAs in COAD, a prognostic model was constructed using the results of the multivariate COX regression. For each patient in the training and testing sets, the risk score was calculated, and patients were divided into high- and low-risk groups based on the median risk score (**Figures 3A, B**). More deaths occurred among patients in the high-risk than in the low-risk group (**Figures 3C**, **D**) and the lncRNA expression of the risk signature showed a significant difference between the groups (**Figures 3E**, **F**). Specifically, expression of the two protective lncRNAs, AC008752.2 and AC012313.5, increased in the low-risk group, while expression of the eight risk lncRNAs increased in the high-risk group (**Figures 3E**, **F**). The KM curve showed that COAD patients in the high-risk group had a worse prognosis than those in the low-risk group (**Figures 3G**, **H**). Moreover, the AUC of the ROC curve further illustrated the accuracy of the risk model. The AUC of the training set for 1-, 2-, and 3- years were 0.800, 0.844, and 0.879, respectively. In the testing set, the AUC values were 0.698, 0.636, and 0.602 for 1-, 2-, and 3- years, respectively (**Figures 3I**, **J**). Moreover, The AUC corresponding to the risk score was higher than that for age, gender and stage in the entire set (**Figure 3K**). We also used the previously published ferroptosis-related lncRNAs signature compared with our signature (19). The results showed that the previously published ferroptosis-related lncRNA signature was significantly related to OS (**Figure S3A**); however, the area under the curve was 0.656 while ours was 0.745 in the overall TCGA group, indicating our signature’s superiority based on cuproptosis-related lncRNAs (**Figures S3B, C**). These findings demonstrate that the risk score is a robust prognostic factor and that the high-risk group is associated with a poor COAD prognosis.

### The risk score could serve as an independent prognostic factor and guide clinical outcome prediction for COAD patients

**Figure 4:**
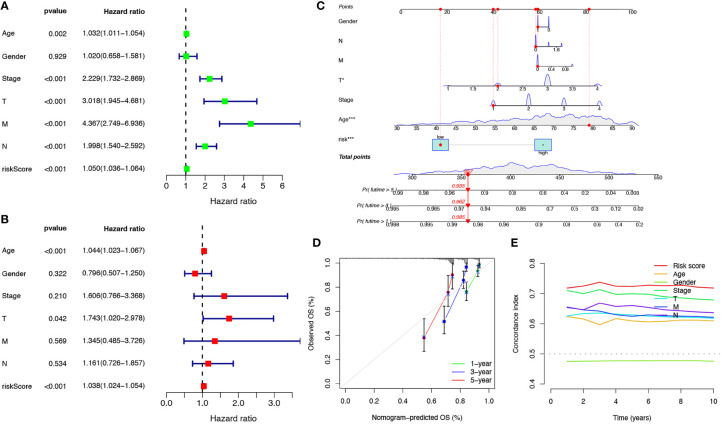
Verification of the independent prognostic ability and clinical predictive ability of the 10 cuproptosis-related lncRNAs signature for COAD in TCGA. Forest plots of univariate **(A)** and multivariate **(B)** Cox regression analyses revealed that risk score could be an independent prognostic factor. The nomogram for both clinical-pathological factors and risk score could predict the probability of survival based on the total points **(C)**. The calibration curves for predicting patients’ OS at 1-year (green line), 3-year (blue line), and 5-year (red line) in the training cohort (left), internal validation cohort (middle), and external validation cohort (right) **(D)**. The C-index curves for assessing the discrimination ability of risk score and other clinical factors at each time point **(E)**. nsP >= 0.05, *P < 0.05, **P < 0.01, ***P < 0.001.

Univariate and multivariate Cox regression analyses were performed to investigate the predictive value of the prognostic signature using cuproptosis-related lncRNAs in COAD. The results revealed that the risk score of the cuproptosis-related lncRNA signature was significantly associated with patient OS and was an independent prognostic factor for COAD patients (**Figures 4A**, **B**). A nomogram combining the clinicopathological features and risk score was created to help clinicians to predict the survival of COAD patients. For example, if the total score of a patient was 357 points, the predicted survival probabilities for this patient were 98.5%, 96.2%, and 93.5% for the next 1, 3, and 5 years, respectively (**Figure 4C**). Calibration curves for OS at 1-, 3-, and 5 years confirmed the predictive power of the prognostic model (**Figure 4D**). It was also shown that the C-index of the prognostic signature was higher than it was for any of the other risk factors (**Figure 4E**). Thus, the model incorporating risk scores and clinical factors can be used to aid COAD patient prognosis.

### Clinical characteristic subgroups and PCA verified the prediction ability and the grouping power of the prognostic signature

**Figure 5:**
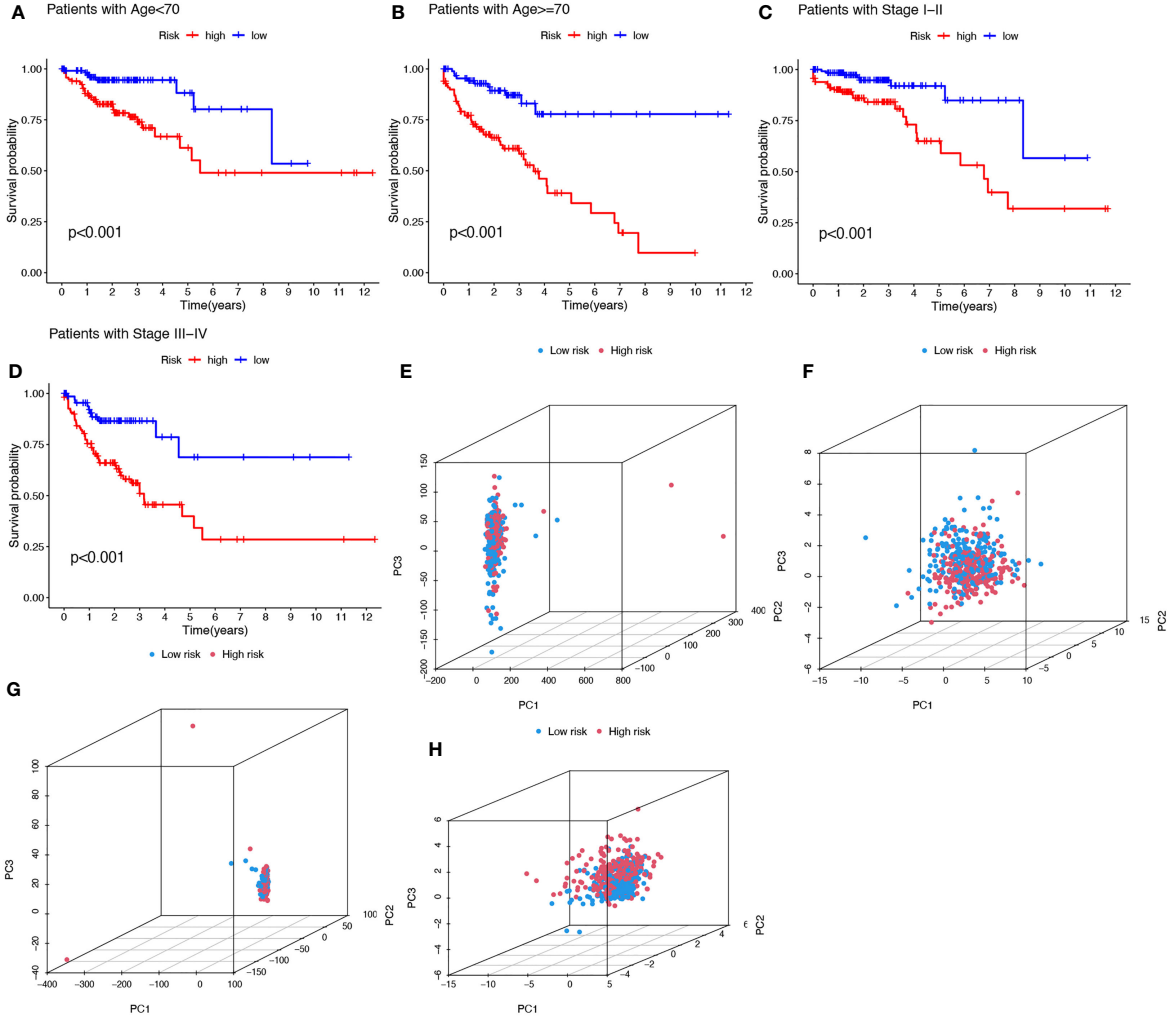
Kaplan-Meier plots depicting subgroup survival analysis stratified by age **(A, B)** and stage **(C, D)**. PCA plots depicted the distribution of samples based on the expression of all genes **(E)**, cuproptosis-related genes **(F)**, lncRNAs **(G)**, and the ten lncRNAs of the prognostic signature **(H)**.

To investigate whether the prediction ability of the prognostic signature was affected by clinical characteristics, patients were divided into subgroups by age or stage, the two most important clinical factors associated with survival. Patients in the high-risk group were found to have a worse prognosis in both the >=70 and< 70 year age subgroups, and in both the stage I-II and stage III-IV subgroups (**Figures 5A-D**). PCA was used to explore the differences between the high- and low-risk groups and no differences were detected based on the expression of all genes (**Figure 5E**), cuproptosis-related genes (**Figure 5F**), or cuproptosis-related lncRNAs (**Figure 5G**). However, COAD patients were separated into two different clusters based on the expression of the ten cuproptosis-related lncRNAs in the signature (**Figure 5H**). Thus, the prognostic signature was able to classify patients with various clinical characteristics into high- and low-risk groups.

### Target lncRNAs are associated with COAD immunity

**Figure 6:**
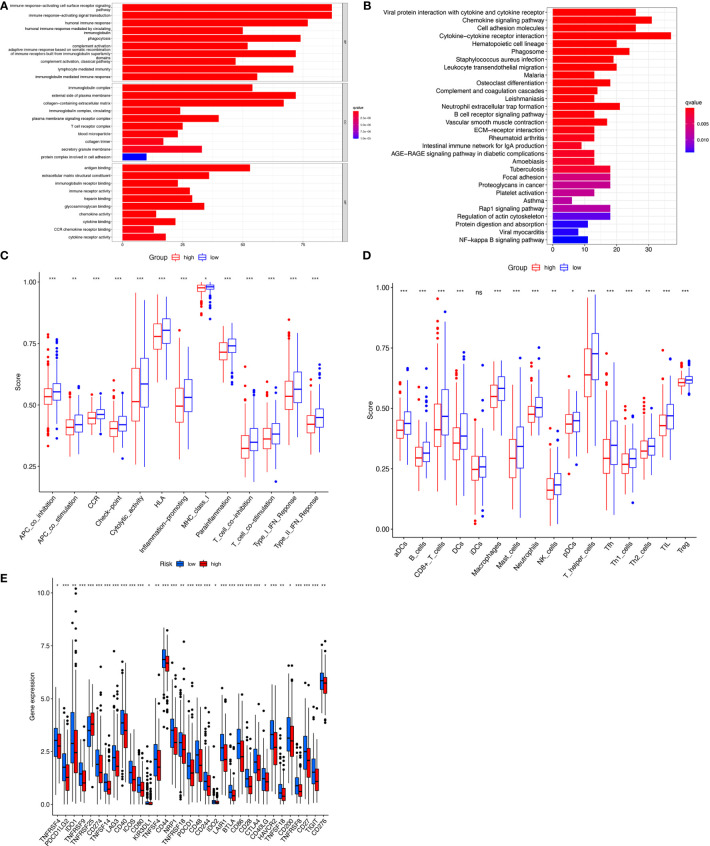
Immunoassay showed that the ten cuproptosis–related lncRNAs were closely related to the immune system. GO and KEGG enrichment analysis of the different expressed genes between two risk groups **(A, B)**. The ssGSEA scores of 13 immune-related functions **(C)** and 16 immune cells **(D)** between different risk groups are displayed in boxplots. Comparison of immune checkpoints between two risk groups **(E)**. nsP >= 0.05, *P < 0.05, **P < 0.01, ***P < 0.001.

To detect differences in biological functions, gene expression of patients in the high- and low-risk groups was compared and 873 differentially expressed genes (DEGs) were obtained (**Table S5**). GO functional enrichment analysis of the DEGs indicated that these genes were highly enriched in immune-activating cell surface receptor signaling pathways, immune response-activating signal transduction, and humoral immune responses in the biological processes group (**Figure 6A**). The KEGG analysis results showed that DEGs were enriched in ‘Cytokine−cytokine receptor interaction’, ‘Chemokine signaling pathway’, ‘Viral protein interaction with cytokine and cytokine receptor’, and ‘Cell adhesion molecules’ (**Figure 6B**). Since the tumor immune microenvironment plays an important role in the occurrence and development of tumors and immune checkpoint inhibitors have revolutionized cancer therapy, the association between risk score and infiltrating tumor immune cells and immune function by ssGSEA were also investigated. Surprisingly, all 13 immune-related functions were significantly different between the high- and low-risk groups, indicating a strong association between immunity and cuproptosis, and between the high-risk phenotype and immunosuppression (**Figure 6C**). Consistent with the immune function, immune cell abundance was significantly lower in the high-risk group (**Figure 6D**). Immune checkpoint gene expression was also compared between the groups and showed that, with the exception of TNFRSF25, the genes tended to be highly expressed in the low-risk group (**Figure 6E**). These findings strongly suggest that cuproptosis may be closely related to tumor immunity.

### Significance of risk models in drug therapy

**Figure 7:**
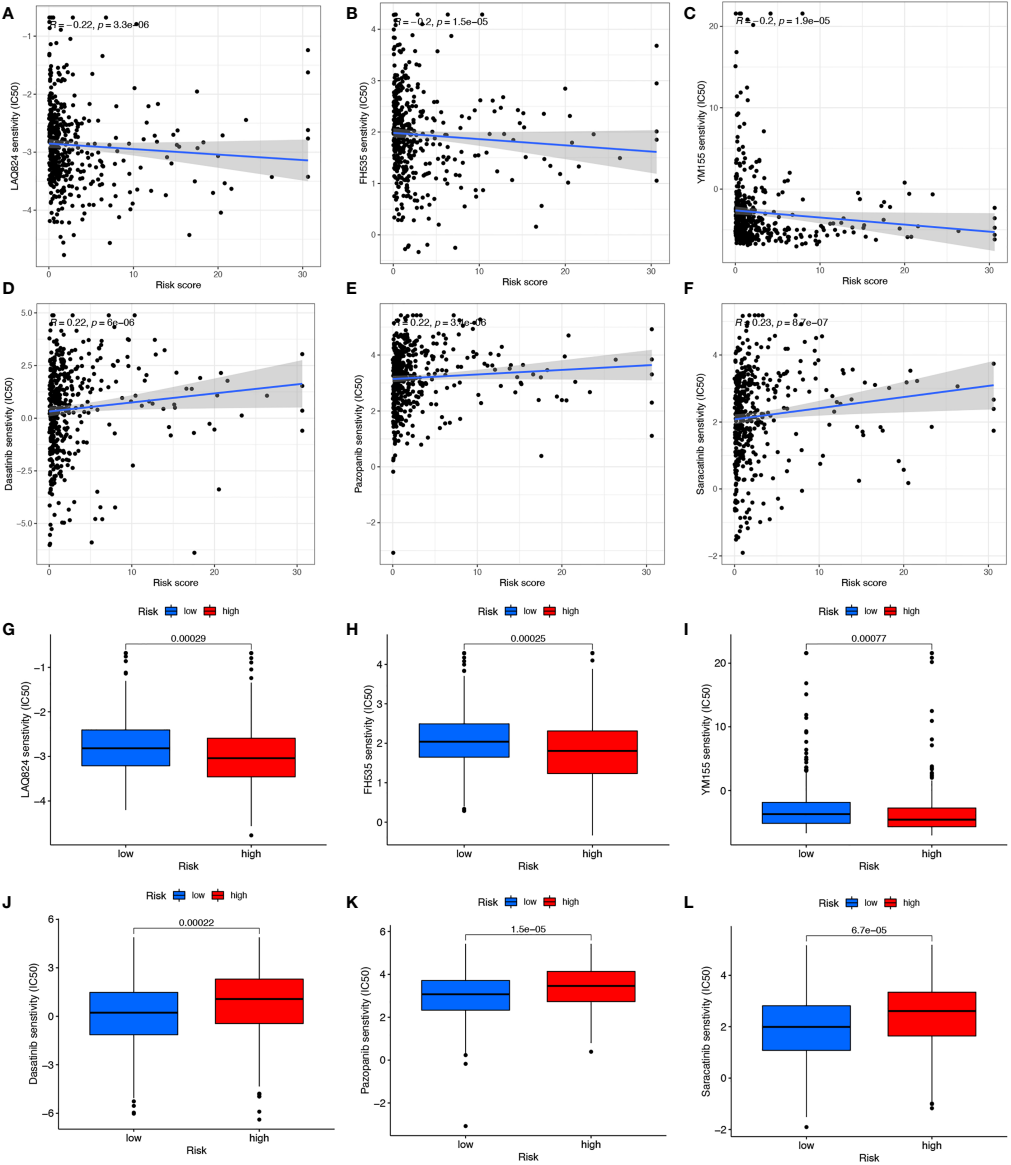
Drug correlation and sensitivity analysis of LAQ824 **(A, G)**, FH535 **(B, H)**, YM155 **(C, I)**, Dasatinib **(D, J)**, Pazopanib **(E, K)** and Saracatinib **(F, L)**.

Responses of COAD patients with different risk scores to various antitumor drugs, including LAQ824, FH535, YM155, Dasatinib, Pazopanib, and Saracatinib, were assessed (**Figures 7A-L**). There were statistically significant differences in drug sensitivity between the high- and low-risk groups. For LAQ824, FH535, and YM155, sensitivity was higher for patients in the high-risk than in the low-risk group (**Figures 7A-C**, **G-I**). For Dasatinib, Pazopanib, and Saracatinib, sensitivity was higher for patients in the low-risk than in the high-risk group (**Figures 7D-F**, **J-L**). The results suggest that this risk model may inform clinical treatment and the prevention of drug resistance in COAD patients.

### Validation of expression of the ten lncRNAs

**Figure 8:**
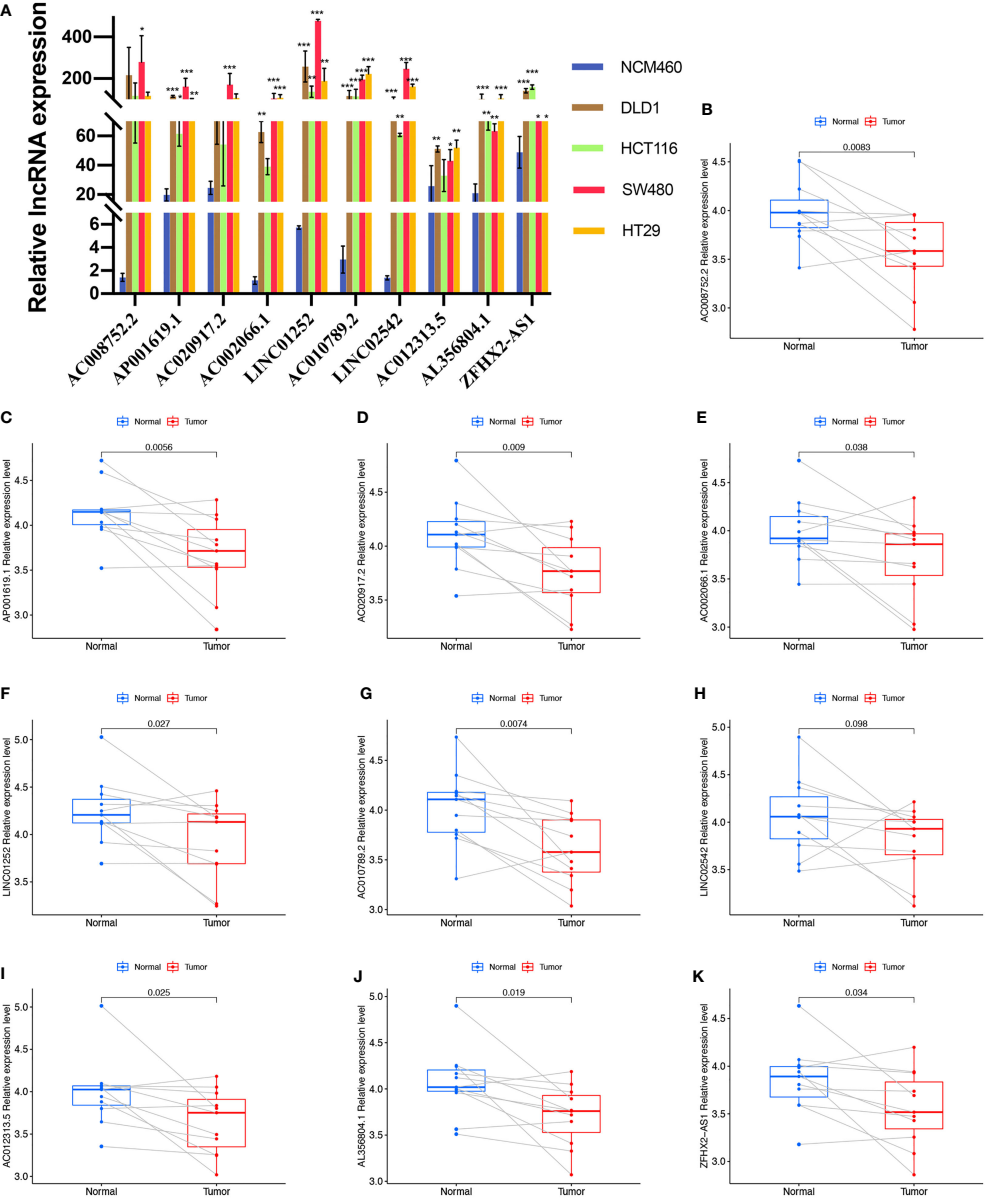
Validation of the expression level of the ten cuproptosis-related lncRNAs in cell lines and human tissues. Expression analysis of AC008752.2, AP001619.1, AC020917.2, AC002066.1, LINC01252, AC010789.2, LINC02542, AC012313.5, AL356804.1 and ZFHX2-AS1 in five cell lines **(A)** and 11 pairs of colon cancer tissue samples **(B-K)**. nsP >= 0.05, *P < 0.05, **P < 0.01, ***P < 0.001.

We first evaluated the expression level of the ten cuproptosis-related lncRNAs in cell lines. Consistent with our hypothesis, the eight risk lncRNAs are highly expressed in colon cancer cell lines (DLD1, HCT116, SW480, HT29) (**Figure 8A**). However, the protective factor AC008752.2 and AC012313.5 were also expressed at a high level in the colon cancer cell lines compared with the normal colon cell line, NCM460 (**Figure 8A**). Expression of the ten cuproptosis-related lncRNAs in clinical samples retrieved from COAD patients in our hospital was further assessed. Interestingly, qRT-PCR results indicated that the expression of AP001619.1, AC020917.2, LINC01252, AC010789.2, AL356804.1, ZFHX2-AS1, AC002066.1, and AC008752.2, AC012313.5 was significantly higher in normal tissue while expression of LINC02542 was similar between normal and tumor tissue (**Figures 8B-K**). Considering the tight association between our risk model and immunity, we hypothesize the immune microenvironment in clinical samples may affect the expression of the lncRNAs. These results suggest that the ten cuproptosis-related lncRNAs, especially the nine differentially expressed lncRNAs, may have regulatory effects during COAD.

### The lncRNAs are regulated by copper

**Figure 9:**
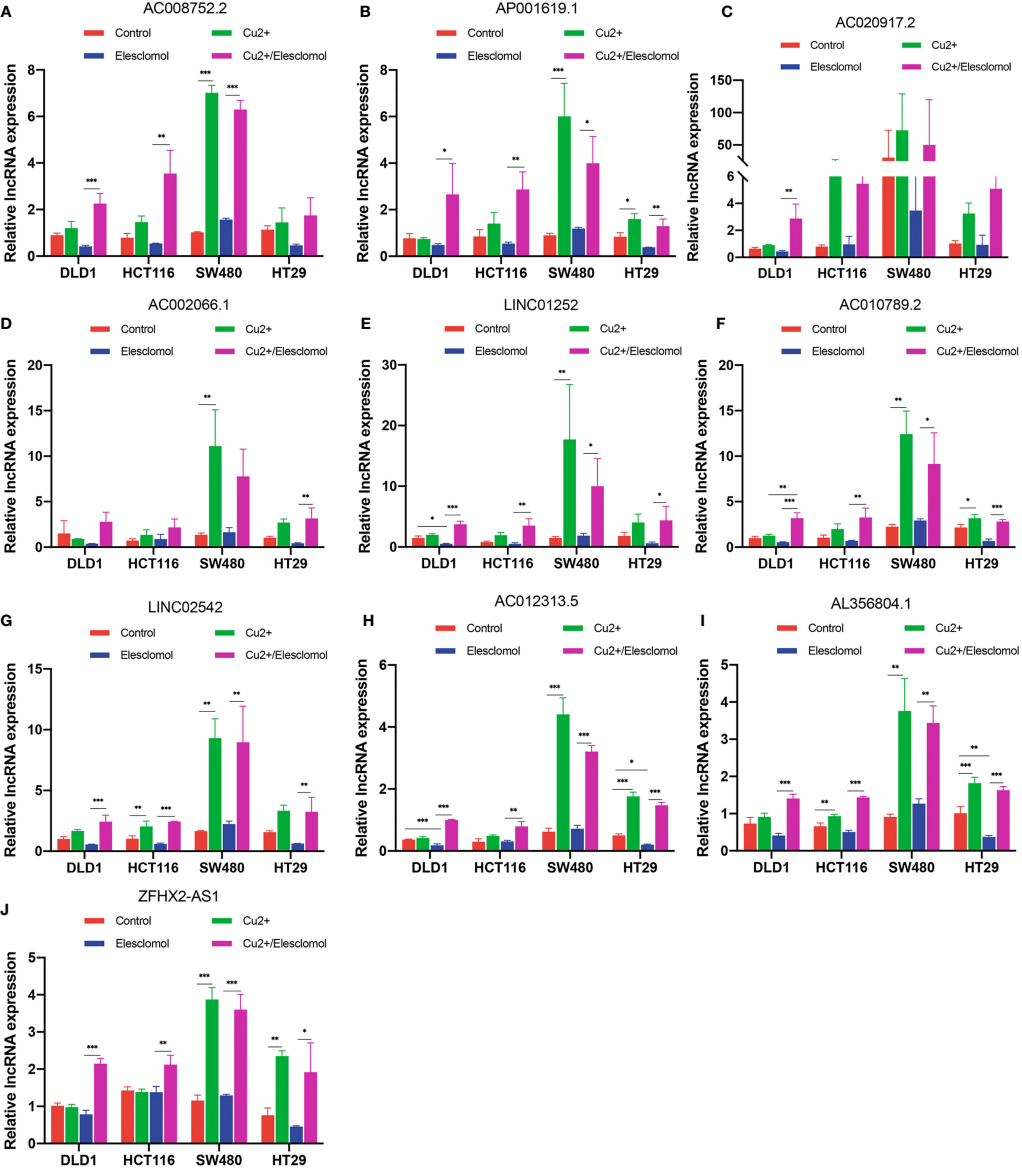
Regulation of cuproptosis in ten lncRNAs. QRT-PCR showing lncRNA expression **(A–J)** of cells treated with indicated drugs for 24 h (n = 3). CuCl_2_ (2μM), elesclomol (20 nM), both CuCl_2_ (2μM) and elesclomol (20 nM). nsP >= 0.05, *P < 0.05, **P < 0.01, ***P < 0.001.

We next evaluated whether copper could affect the expression of these 10 lncRNAs. Elesclomol is a copper ionophore that shuttles copper into the cell. We treated colon cancer cell lines with copper chloride (2μM) or elesclomol (20nM) or combination of elesclomol and copper chloride for 24h and observed the expression of ten lncRNAs in four colon cancer cell lines using qRT-PCR. In the presence of elesclomol, almost all 10 lncRNAs were significantly upregulated in colon cancer cell lines after cuproptosis induced by exogenous introduction of copper ions (**Figures 9A-J**). The results confirm that these lncRNAs play important roles in cuproptosis in COAD.

## Discussion

The concept cuproptosis was coined in 2022 March and little is reported in the literature on this issue to date. Cuproptosis is a copper-dependent form of regulated cell death driven by excessive lipoylated dihydrolipoamide S-acetyltransferase (DLAT) lipoylation. Accumulating evidence have shown that the development of tumors is always related to programmed cell death (20–22). Bian Z et al. constructed a cuproptosis-related prognostic gene signature in clear cell renal cell carcinoma, confirming the importance of cuproptosis in tumor development and progression (23). In-depth research in cuproptosis helps to facilitate new understandings of the pathogenesis of tumors.

The function and regulatory effect of lncRNAs has been increasingly recognized in colon cancer. Yue et al. reported that lncRNA CYTOR played an important role in colon cancer metastasis and that the positive feed-forward circuit of CYTOR-β-catenin may be a useful therapeutic target in the antimetastatic strategy (24). LINC01123 could act as an oncogene and promote colon cancer malignancy and chemoresistance (25). LINC01123 depletion hinders colon cancer cell proliferation. This indicates that lncRNAs may be useful in colorectal cancer prognosis. A role for ferroptosis-related lncRNAs in colon cancer has also been reported (19, 26). Wu et al. constructed a 4-ferroptosis-related signature, which was found to be a promising biomarker to predict clinical outcomes and therapeutic responses among colon cancer patients (26). However, no studies to date have assessed the role of cuproptosis-related lncRNAs in colon cancer. Thus, it is necessary to establish a risk signature based on cuproptosis-related lncRNAs.

In this study, we screened out lncRNAs co-expressed with cuproptosis genes and constructed a ten cuproptosis-related lncRNAs signature which may be a potential biomarker for colon cancer diagnosis and prognosis stratification. We first examined 318 prognostic lncRNAs associated with cuproptosis in TCGA-COAD data set using univariate Cox regression analysis. Next, 10 cuproptosis-related lncRNAs (AC008752.2, AP001619.1, AC020917.2, AC002066.1, LINC01252, AC010789.2, LINC02542, AC012313.5, AL356804.1 and ZFHX2-AS1) were identified *via* LASSO regression and multivariate Cox regression analysis. The prognostic signature was then constructed. Kaplan-Meier survival curve, ROC curve and calibration curve evaluated the predictive accuracy of the cuproptosis-related signature in COAD patients. Finally, it was determined that the risk signature based on these ten lncRNAs was an independent factor for colon cancer and was of great significance as a guide in clinical practice.

Previous study reported AP001619.1 was incorporated into a prognostic ceRNA signature in colon cancer (27). Li N et al. reported that AC012313.5 participated in prognostic ferroptosis-associated lncRNAs signature in CRC patients (28). Of interest, the fact that AC012313.5 was associated with both cuproptosis and ferroptosis appears to hint the view that cuproptosis is a type of ferroptosis-associated cell death. cancer cells with high basal ROS level are vulnerable to disrupted mitochondrial respiration caused by excessive copper accumulation while ferroptosis, characterized by the elevated lipid peroxidation, was a form of ROS-related cell death (29). It is also reported in the literature that copper could react with disulfiram to induce ferroptosis in HCC (30). More importantly, cancer cells with therapeutic resistance frequently acquire sensitivity to ferroptosis (31). The close connection between cuproptosis and ferroptosis strongly suggest that cuproptosis is a promising target to bring new hope for future antitumor treatment.

Studies indicate that the prognostic value of adaptive immune cell infiltration is superior to classical tumor invasion criteria, including grade, stage, and metastatic status (32, 33). In addition, the degree of immune infiltration and the levels of immune mediators are related to colon cancer prognosis (34). Research on the efficacy of immune checkpoint inhibitor (ICI) therapy in mismatched repair-deficient and microsatellite instability high (dMMR-MSI-H) colon cancer tumors show promising results. A phase I clinical trial of MDX-1106, an anti-PD-1 antibody, in patients with a variety of treatment-resistant tumors, including one patient with colorectal cancer, culminated in the patient achieving a durable complete response (35). The use of nivolumab in CheckMate-142 showed a 31% objective response rate and a 73% twelve-month OS rate in treatment-intolerant dMMR-MSI-H colorectal cancer, including durable responses and disease control as well as long-term survival (36).

In this study, a large number of immune-related biological processes were enriched between the two risk groups and significant differences between the two risk groups in immune cell abundance, immune function, and immune escape were found. Therefore, it can be reasonably assumed that cuproptosis may be critically involved in tumor immunity. TNFRSF25 is a member of the TNF receptor superfamily (TNFRSF) that acts as the functional receptor of TL1A (37). In this study, TNFRSF25 expression was higher in the high-risk than in the low-risk group. TNFRSF25 signaling provides co-stimulatory signals for activated lymphocytes and subsequently generates protective antitumor CD8+ CTL responses (38). The expression levels of immune checkpoints (CTLA-4, PD-1 and PD-L1) were investigated between the two risk groups and the low-risk COAD patients had significantly higher expression of CTLA4, PD-1 and PD-L1 than the high-risk patients. This implies that these patients may benefit from an immune checkpoint blockade strategy (39), which might improve the prognosis of low-risk patients by enhancing their immunoreactivity or inducing cuproptosis. Immune cell infiltration, immune function, and the expression of immune checkpoint genes lead to individual differences in the prognosis of patients with colon adenocarcinoma. This suggests that using the model to predict the risk scores of patients with colon cancer can reflect the effect of ICI and the immune response rate to some extent. Based on the characteristics of the two risk groups, drug sensitivity analysis was conducted, and each group was found to be sensitive to three representative drugs, which may help to achieve better overall outcomes.

This study has some limitations. First, the risk signature was created using public data and lacks novel clinical samples and data. Second, because no other databases have available lncRNA expression and clinical data, the model had to be validated in one database. In addition, due to insufficient samples, qRT-PCR was only performed on 11 pairs of clinical samples to verify the expression of ten cuproptosis-related lncRNAs comprising this signature. Thus, the function of this signature must be validated in clinical research.

## Conclusion

In summary, a ten cuproptosis-related lncRNAs signature associated with COAD patient prognosis was identified. The correlation between this signature and the immune landscape was preliminarily ascertained and the relationship between chemosensitivity and immune checkpoint inhibitors was assessed. The findings of this study may provide new research strategies for exploring the mechanisms of cuproptosis and expand current insights into therapeutic approaches for COAD patients.

## Data availability statement

Publicly available datasets were analyzed in this study. This data can be found here: the TCGA database (https://portal.gdc.cancer.gov/).

## Ethics statement

The studies involving human participants were reviewed and approved by Medical Ethics Committee of the Second Affiliated Hospital of Zhejiang University School of Medicine. The patients/participants provided their written informed consent to participate in this study.

## Author contributions

This research was conducted in collaboration with all authors. MX and JM performed the data curation and analysis. JJW and QZ analyzed and interpreted the results. JWW drafted and reviewed the manuscript. All authors contributed to the article and approved the submitted version.

## Funding

This study was supported by the National Natural Science Foundation of China (Grant number: 81672364, Grant number: 81871917).

## Acknowledgments

The authors gratefully acknowledge the Cancer Genome Atlas (TCGA) database, which made the data available.

## Conflict of interest

The authors declare that the research was conducted in the absence of any commercial or financial relationships that could be construed as a potential conflict of interest.

## Publisher’s note

All claims expressed in this article are solely those of the authors and do not necessarily represent those of their affiliated organizations, or those of the publisher, the editors and the reviewers. Any product that may be evaluated in this article, or claim that may be made by its manufacturer, is not guaranteed or endorsed by the publisher.

## Supplementary material

Supplementary Figure 1 Workflow of the construction and validation of this prognostic signature

Supplementary Figure 2 Associations between cuproptosis-related lncRNAs expression and survival. **(A-J)** The Kaplan-Meier curves showed overall survival of patients with high(red) or low(blue) cuproptosis-related lncRNAs expression in the training set. p< 0.05 in the two-sided log-rank test was considered statistically significant.

Supplementary Figure 3 Comparison with the previously published lncRNAs signature. **(A)** Kaplan-Meier curve analyses showed high-risk group was correlated with poor prognosis in COAD according to the previous study. **(B)** The accuracy of the previously published model **(B)** and our signature **(C)** was verified using ROC analysis in TCGA database.
