# Supplementary figures and images for: Construction and validation of a cuproptosis-related lncRNA signature as a novel and robust prognostic model for colon adenocarcinoma
Source: Front Oncol. 2022 Jul 28;12:961213. doi: 10.3389/fonc.2022.961213 (PMC9367690; doi:10.3389/fonc.2022.961213)

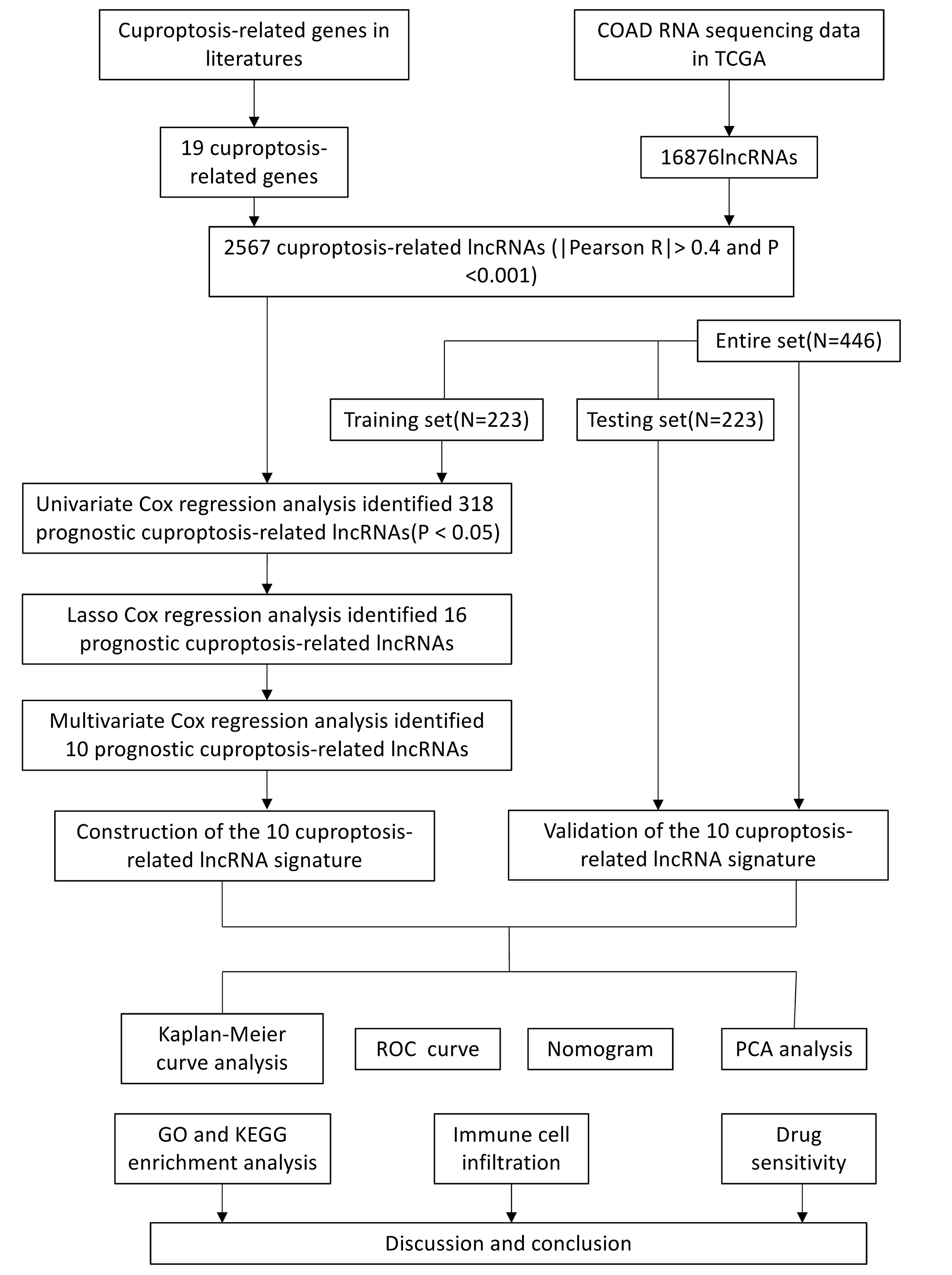

Supplement: Supplementary Figure 1 — Workflow of the construction and validation of this prognostic signature [file Image_1.tiff]

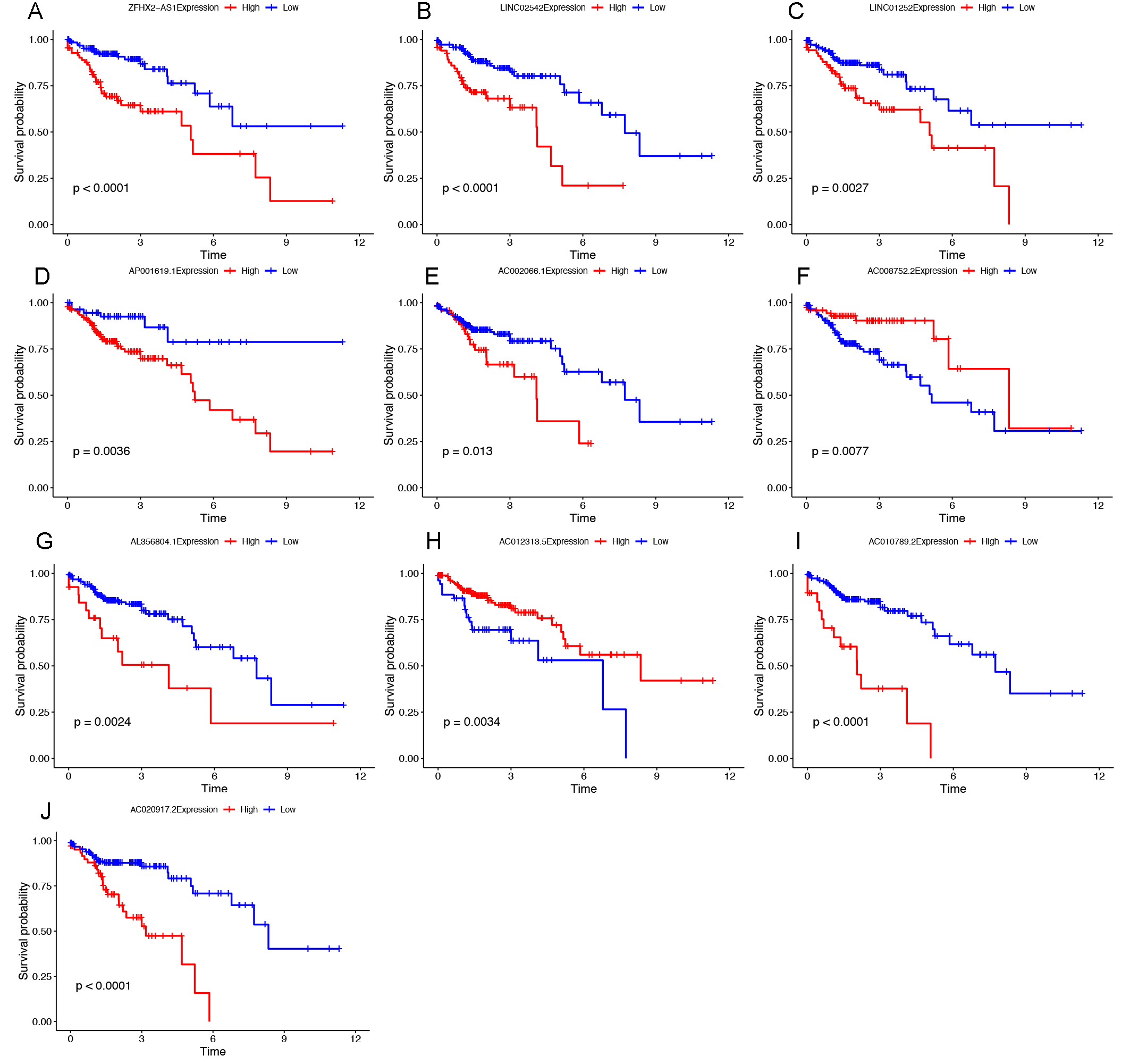

Supplement: Supplementary Figure 2 — Associations between cuproptosis-related lncRNAs expression and survival. (A-J) The Kaplan-Meier curves showed overall survival of patients with high(red) or low(blue) cuproptosis-related lncRNAs expression in the training set. p< 0.05 in the two-sided log-rank test was considered statistically significant. [file Image_2.tiff]

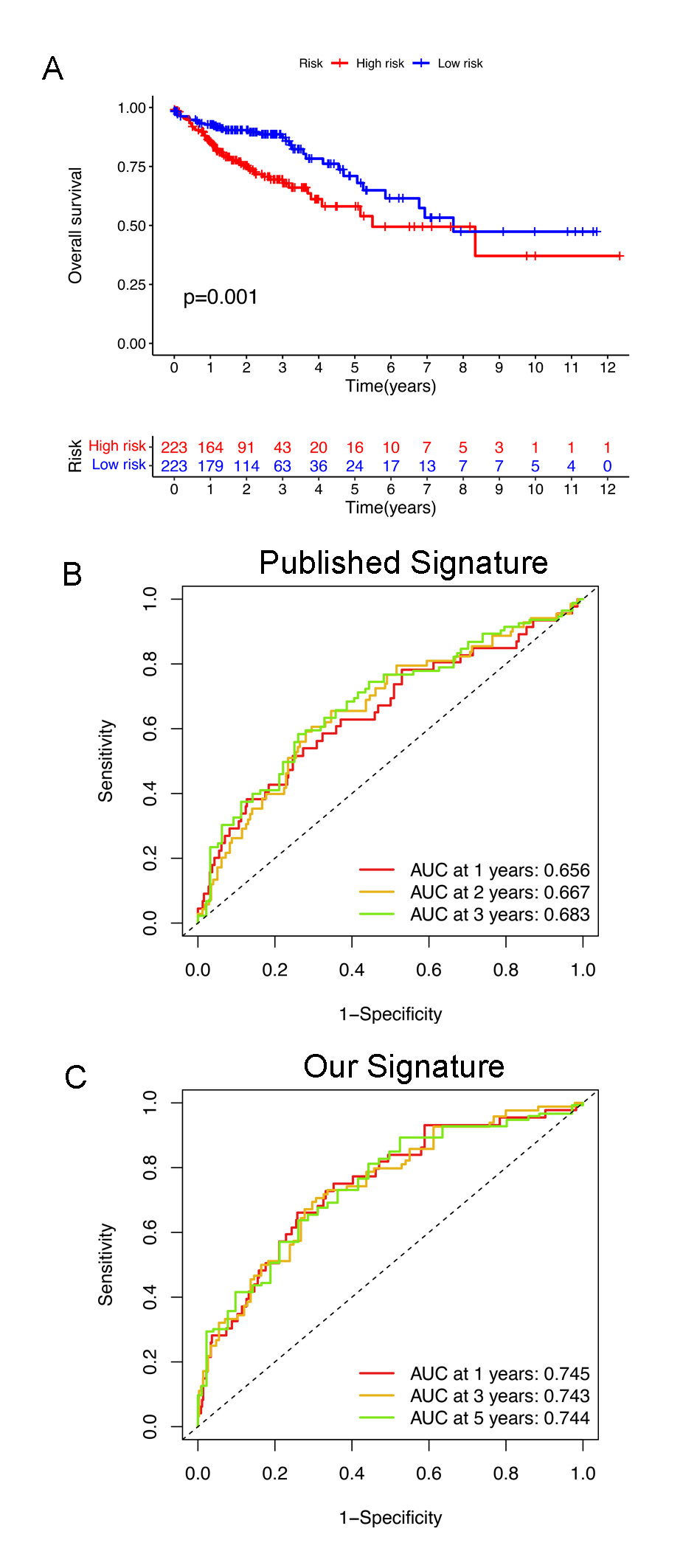

Supplement: Supplementary Figure 3 — Comparison with the previously published lncRNAs signature. (A) Kaplan-Meier curve analyses showed high-risk group was correlated with poor prognosis in COAD according to the previous study. (B) The accuracy of the previously published model (B) and our signature (C) was verified using ROC analysis in TCGA database. [file Image_3.tiff]
