# Supplementary material for: Construction and validation of a cuproptosis-related lncRNA signature as a novel and robust prognostic model for colon adenocarcinoma
Source: Front Oncol. 2022 Jul 28;12:961213. doi: 10.3389/fonc.2022.961213 (PMC9367690; doi:10.3389/fonc.2022.961213)
Supplement: Supplementary file 6 [file Table_3.docx]

**Table.S3** the number of co-expressed lncRNAs for each cuproptosis gene

| Cuproptosis-related gene | the number of co-expressed lncRNAs |
| --- | --- |
| ATP7A | 1665 |
| ATP7B | 178 |
| CDKN2A | 0 |
| DBT | 300 |
| DLAT | 2 |
| DLD | 1 |
| DLST | 1 |
| FDX1 | 1 |
| GCSH | 2308 |
| GLS | 2083 |
| LIAS | 61 |
| LIPT1 | 53 |
| LIPT2 | 2 |
| MTF1 | 325 |
| NFE2L2 | 25 |
| NLRP3 | 376 |
| PDHA1 | 0 |
| PDHB | 1 |
| SLC31A1 | 0 |
